# Pericytes and Neurovascular Function in the Healthy and Diseased Brain

**DOI:** 10.3389/fncel.2019.00282

**Published:** 2019-06-28

**Authors:** Lachlan S. Brown, Catherine G. Foster, Jo-Maree Courtney, Natalie E. King, David W. Howells, Brad A. Sutherland

**Affiliations:** School of Medicine, College of Health and Medicine, University of Tasmania, Hobart, TAS, Australia

**Keywords:** pericytes, brain, neurovascular, neurological disease, cerebral blood flow, blood brain barrier

## Abstract

Pericytes are multi-functional cells embedded within the walls of capillaries throughout the body, including the brain. Pericytes were first identified in the 1870s, but little attention was paid to them during the following century. More recently, numerous vascular functions of pericytes have been identified including regulation of cerebral blood flow, maintenance of the blood-brain barrier (BBB), and control of vascular development and angiogenesis. Pericytes can also facilitate neuroinflammatory processes and possess stem cell-like properties. Pericytes form part of the neurovascular unit (NVU), a collection of cells that control interactions between neurons and the cerebral vasculature to meet the energy demands of the brain. Pericyte structure, expression profile, and function in the brain differ depending on their location along the vascular bed. Until recently, it has been difficult to accurately define the sub-types of pericytes, or to specifically target pericytes with pharmaceutical agents, but emerging techniques both *in vitro* and *in vivo* will improve investigation of pericytes and allow for the identification of their possible roles in diseases. Pericyte dysfunction is increasingly recognized as a contributor to the progression of vascular diseases such as stroke and neurodegenerative diseases such as Alzheimer’s disease. The therapeutic potential of pericytes to repair cerebral blood vessels and promote angiogenesis due to their ability to behave like stem cells has recently been brought to light. Here, we review the history of pericyte research, the present techniques used to study pericytes in the brain, and current research advancements to characterize and therapeutically target pericytes in the future.

## History of Pericyte Research

**FIGURE 1:**
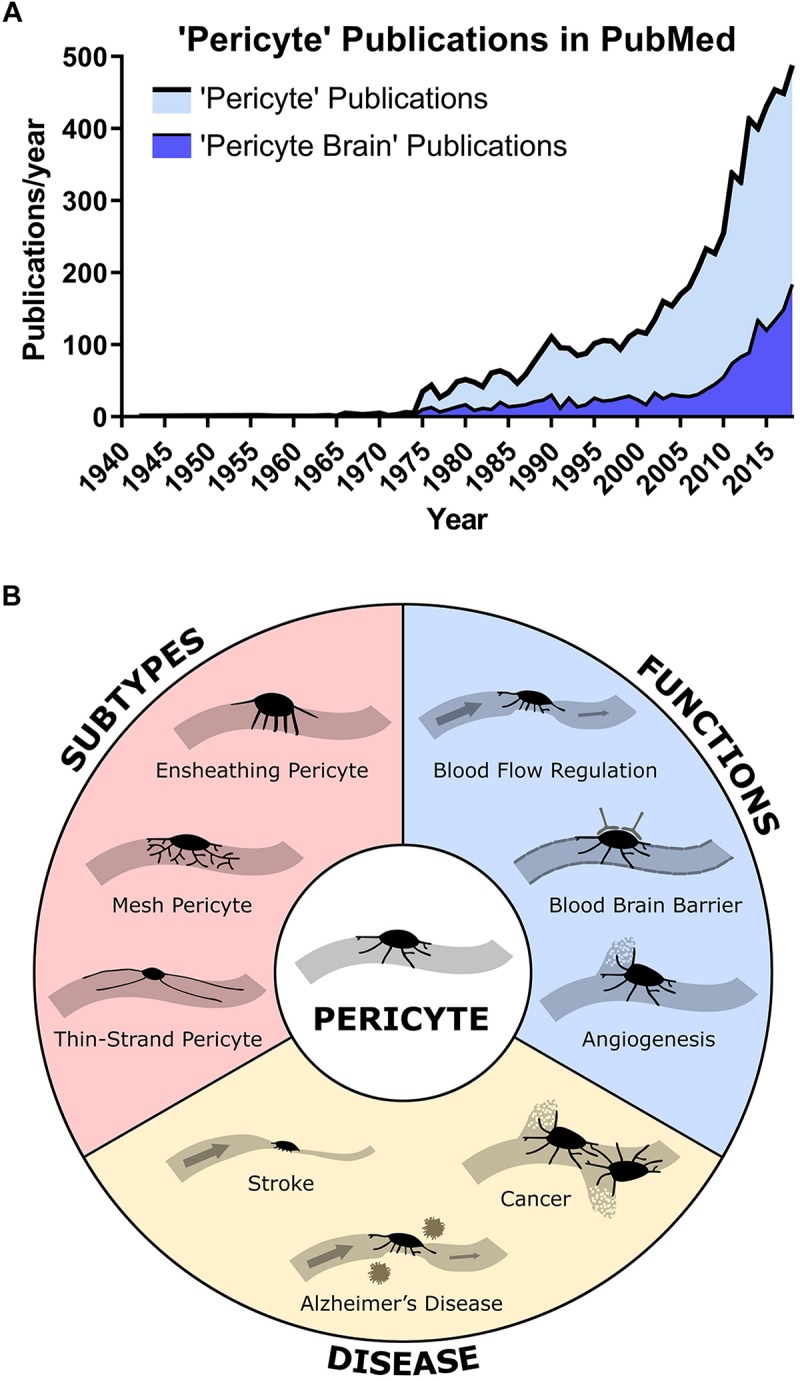
**(A)** Publications found in PubMed with the search term “Pericyte” or “Pericyte Brain” by year showing a rapid increase in publications in the 21st century. About 25% of all pericyte papers are focused on the brain. **(B)** Overview of main pericyte sub-types, their functions, and roles in disease.

Pericytes were first identified in the late 19th century by Eberth, then Rouget, as spatially isolated cells associated with the capillary wall (Attwell et al., 2016). They were found embedded within the basement membrane, both on straight sections and at branch points of capillaries, with projections extending from the soma to wrap around the underlying vessel. In the 1920s, Zimmerman named these cells pericytes, stating that he included various cell morphologies under the definition of pericyte, including their transitional forms to vascular smooth muscle cells (VSMC) at the arteriolar end (Zimmermann, 1923). This definition is used broadly in research investigating pericyte function and has led to debate over what constitutes a pericyte (Hill et al., 2015; Attwell et al., 2016). Throughout the 20th century, there was relatively little research into pericytes. With advancing technologies and more accurate techniques to identify and study pericytes, research into pericyte function has increased exponentially over the last 15 years (Figure 1A). Numerous studies have reported novel functions for pericytes in different organs of the body, including the heart (Avolio and Madeddu, 2016) and the brain (Sweeney et al., 2016), with 25% of all papers on pericytes focused on the brain (Figure 1A). Current research largely focuses on characterizing pericyte mechanisms of function, defining pericyte sub-classes (Figure 1B), their roles in health and disease, and their potential as a therapeutic target to treat disease.

## Current State of Knowledge on Pericytes in the Brain

### Pericytes in the Neurovascular Unit

**FIGURE 2:**
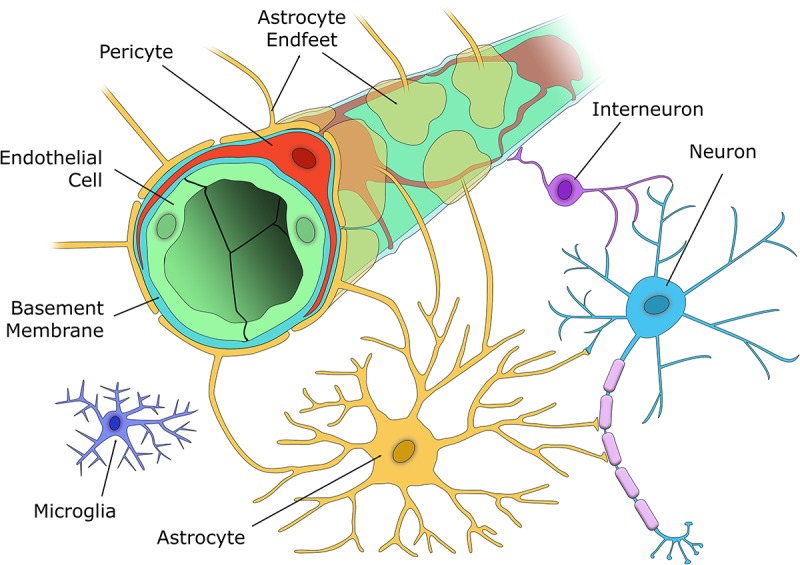
The cellular components of the NVU. The capillary wall contains a single EC layer (green) connected by tight junctions that form the blood-brain barrier along with pericytes (red), that are embedded within the capillary basement membrane (light blue), and nearby astrocyte endfeet (yellow). Excitatory neurons (blue) synapse with both vasoactive interneurons (purple), and astrocytes (yellow), who in turn signal to the capillary to alter blood flow according to the metabolic demands of that brain region. Microglia (indigo) are located in the brain parenchyma and respond to any aversive stimuli to protect the brain.

The brain is one of the most energy-demanding organs in the body. Despite only accounting for 2% of the body’s mass, over 20% of cardiac output is distributed to the brain to meet oxygen and glucose requirements at rest (Xing et al., 2017). To deliver blood flow to neuronal and glial cells, the mammalian brain has evolved the neurovascular unit (NVU), a complex unit of cells that connects the brain parenchyma to the cerebral vasculature. The NVU consists of brain parenchymal cells including excitatory neurons, inhibitory interneurons, astrocytes, and microglia interacting with vascular cells including pericytes (on capillaries), VSMC (on arterioles and arteries), and endothelial cells (EC) (McConnell et al., 2017; Figure 2). The NVU plays important roles in maintaining brain function, particularly the regulation of cerebral blood flow (CBF) and the formation of the blood-brain barrier (BBB). Pericytes are central to NVU function as they are located at the interface between the brain parenchyma and the blood vessels, and so can act as chemical sensors to enable communication between the two groupings of cells.

### Functions of Pericytes

#### Cerebral Blood Flow

The mammalian brain has evolved a mechanism for regional control of CBF known as neurovascular coupling, which ensures a rapid increase in the amount of CBF directed to active neurons (Attwell et al., 2010). Neurovascular coupling is controlled by the cells within the NVU, which includes pericytes.

Within the brain, pericytes can actively relax or contract to change CBF in response to localized changes in neuronal activity (Hall et al., 2014; Mishra et al., 2016; Kisler et al., 2017b; Cai et al., 2018). Pericytes possess contractile proteins including alpha-smooth muscle actin (α-SMA), tropomyosin, and myosin which give rise to their contractile ability (Rucker et al., 2000; Alarcon-Martinez et al., 2018). However, it appears that only a subset of pericytes perform this role, namely the ensheathing pericytes at the arteriolar end of the capillary bed which express higher amounts of α-SMA compared to thin-strand pericytes in the middle and at the venous end of the capillary, though this remains controversial (Alarcon-Martinez et al., 2018). Still, it is important to note that red blood cells deform the walls of capillaries when passing through (Jeong et al., 2006), so any change to tone or rigidity of mid-capillary or venule end pericytes may also affect CBF by altering the stiffness of the capillary wall, thereby changing capillary transit time (Attwell et al., 2016).

#### Vascular Development and Maintenance

Correct development of the cerebral microvasculature is essential for neuronal function and pericytes play a critical role in both development and maintenance of cerebral microcirculation. In adult-viable pericyte-deficient mice, pericyte loss leads to vascular dysfunction via a reduction in brain microcirculation, diminished capillary perfusion, loss of blood flow responses to brain activation, and BBB breakdown associated with brain accumulation of neurotoxic serum molecules (Bell et al., 2010). EC use growth factors such as angiopoietin 1, transforming growth factor beta (TGF-β) and platelet-derived growth factor-BB (PDGF-BB) to direct pericytes to migrate to new vessels in order to stabilize the vascular wall (Ribatti et al., 2011). At the capillary level, pericytes appear to control the cell cycle of EC, as well as directly contribute to the formation of the basement membrane (Bergers and Song, 2005). Pericytes also secrete angiogenic-promoting factors such as vascular endothelial growth factor and neurogenic locus notch homolog protein (NOTCH) 3 to activate angiogenic processes in the adult central nervous system (Ribatti et al., 2011).

#### The Blood-Brain Barrier

The BBB is a diffusion barrier vital for preventing toxic material from the circulation entering the brain. Function of the BBB is reliant on non-fenestrated EC that form the blood vessel wall and is supported by both pericytes and astrocytes. Pericytes can modulate and maintain the BBB through the release of signaling factors to determine the number of EC tight junctions and direct the polarization of astrocyte endfeet (Armulik et al., 2010). A reduction in pericyte numbers can cause a loss of tight junctions between EC, leading to increased BBB permeability (Sengillo et al., 2013). In addition, pericytes can control the movement of substances between the blood stream and the brain parenchyma, including the vascular clearance of toxic species out of the brain (Ma et al., 2018).

#### Neuroinflammation

Neuroinflammation is primarily driven by microglia, astrocytes, and infiltrating leukocytes, though pericytes are also capable of performing immune cell functions (Jansson et al., 2014). Pericytes can phagocytose other cells, respond to and express inflammatory molecules and cytokines, and present antigens to immune cells (Rustenhoven et al., 2017). Exposure of pericytes to cytokines such as interleukin-1 beta (IL-1β) and tumor necrosis factor alpha (TNF-α) triggers the release of inflammatory molecules and matrix metalloprotease 9 (MMP9), leading to BBB breakdown (Herland et al., 2016). In addition, it has been shown that expression of apolipoprotein E, a major genetic risk factor for Alzheimer’s disease (Kim et al., 2009), or a lack of murine apolipoprotein E can lead to vascular dysfunction and BBB breakdown by activating the pro-inflammatory CypA-nuclear factor-κB-MMP9 pathway in pericytes (Bell et al., 2012). As a result, pericytes are able to recruit immune cells and enable their extravasation into the brain (Rustenhoven et al., 2017). In transgenic pericyte-deficient mice there is reduced leukocyte trafficking across the microvasculature, suggesting that pericytes play a role in leukocyte recruitment into the brain (Wang et al., 2006).

#### Stem Cell Potential

Pericytes are often considered to be similar to mesenchymal stem cells and both *in vitro* and *in vivo* studies have shown they can differentiate into multiple cell types including angioblasts, neural progenitors, vascular cells, and microglia (Ozen et al., 2014; Nakagomi et al., 2015). The exact mediators that control differentiation of pericytes are still under investigation but pericyte pluripotency can be utilized as a target for therapeutic intervention for a number of diseases. An interesting example of pericyte pluripotency is from the dental pulp where pericytes could be differentiated into both glial and neuronal cell types (Farahani et al., 2019). However, this example uses peripheral pericytes and there is increasing evidence at the single-cell transcriptomic level that there are molecular and functional differences between brain-derived pericytes and pericytes residing in the periphery (Vanlandewijck et al., 2018).

### Paracrine Interactions of Pericytes

Endothelial cells and pericytes communicate by juxtacrine and paracrine signaling through mediators such as TGF-β, angiopoietin 1/2, vascular endothelial growth factor (VEGF), and PDGF-BB (Sweeney et al., 2016). These factors are important in directing vessel development and maturation and formation of the BBB, as well as pericyte survival and contractility (Armulik et al., 2011). The importance of pericyte-EC interactions can be seen in pericyte depletion animal models, such as platelet-derived growth factor receptor beta (PDGFR-β) knockdown mice, that display abnormal vascular growth and extensive EC apoptosis (Hellstrom et al., 2001). Pericytes communicate with each other, allowing the propagation of signals up or down the capillary, which can assist in the control of blood flow through the capillary network (Peppiatt et al., 2006). Astrocytes and pericytes can interact to alter BBB permeability through astrocyte-secreted apolipoprotein binding to the low density lipoprotein receptor-related protein 1 receptor (LRP-1) on pericytes (Ma et al., 2018), and modulate CBF through astrocyte secretion of prostaglandin E_2_ binding to pericyte EP4 receptors (Mishra et al., 2016). Neurons may interact with pericytes through the release of neurotransmitters to induce pericyte relaxation (glutamate) or contraction (noradrenaline) thus altering vascular tone (Peppiatt et al., 2006; Hall et al., 2014). It is also possible that pericytes may influence neuronal function through the release of neurotrophic factors (Shimizu et al., 2012). Overall, the interplay between pericytes and other cell types of the NVU is complex but enables precise control over CBF and BBB permeability.

## Pericyte Dysfunction in Neurological Disease

Aberrant pericyte function and/or pericyte deficiency accompanied by vascular dysfunction has been observed in several disease states. A recent publication thoroughly reviews evidence of pericyte dysfunction in a diverse number of neurological disorders including stroke, AD, glioma and other tumors, traumatic brain injury, migraine, epilepsy, spinal cord injury, diabetes, Huntington’s disease, multiple sclerosis, radiation necrosis, and amyotrophic lateral sclerosis (Cheng et al., 2018). A short description of pericyte functions in stroke, AD, and tumor formation is provided below.

### Ischemic Stroke

Ischemic stroke involves the disruption of blood supply to the brain and can cause extensive neuronal death. While reopening of the blocked artery has been the predominant focus of ischemic stroke therapy, there has been limited attention given to the restoration of capillary blood flow. Multiple studies have shown that following a stroke, pericytes constrict capillaries and die in rigor causing the capillary to be clamped shut leading to a long-lasting restriction of blood flow (no-reflow). These changes to pericytes post-stroke appear to be driven by an influx of intracellular calcium and oxidative stress (Yemisci et al., 2009; Hall et al., 2014). In addition, ischemia leads to the release of MMP9 by pericytes, which interrupts the tight junctions between EC and the binding of astrocyte endfeet to the vascular wall, increasing BBB permeability (Underly et al., 2017). Therefore, pericytes have been identified as an attractive therapeutic target to prevent ongoing microvascular-mediated restriction of CBF and opening of the BBB following stroke.

### Alzheimer’s Disease

Alzheimer’s disease (AD), a prevalent neurodegenerative disorder, is characterized by the accumulation of amyloid beta (Aβ) to form plaques and hyperphosphorylated tau to form neurofibrillary tangles. However, it is becoming increasingly recognized that vascular dysfunction through restriction of CBF and impairment in the BBB could contribute to and even precede AD onset and progression. It is hypothesized that Aβ may cause pericyte death, leading to reduced CBF and loss of BBB integrity, which may in turn accelerate neuronal degeneration and Aβ plaque build-up (Winkler et al., 2014). *In vitro* studies have confirmed Aβ can cause pericyte toxicity (Wilhelmus et al., 2007) and both rodent and human studies have shown that pericyte loss occurs in AD (Sagare et al., 2013; Sengillo et al., 2013). Pericytes can endocytose perivascular debris, giving them a vital role in the clearance of molecules such as Aβ (Ma et al., 2018) and may have protective effects in AD. A recent publication demonstrates that soluble PDGFR-β is a cerebrospinal fluid biomarker of pericyte injury during early cognitive impairment and correlates with BBB disruption independent of Aβ and tau (Nation et al., 2019). Therefore, identifying ways to prevent pericyte loss in AD is important given that cerebrovascular changes such as BBB dysfunction and reduced cerebrovascular density can occur early in the disease. A recent review that focusses on BBB physiology more comprehensively details pericyte changes in AD (Sweeney et al., 2019).

### Tumor Formation

Tumor development relies on fast and efficient vascularisation to match growth, but the abnormal branching, disorganized networks, uneven basement membranes, and lack of pericytes favors non-productive angiogenesis and metastasis (Barlow et al., 2012). Therefore, delivery of pericyte therapy to the tumor can allow vascular normalization, preventing metastasis and improving the efficacy of chemo- or radio-therapy (Meng et al., 2015). However, many tumor types rely on angiogenesis for growth, for which pericytes are critical, and so the inhibition of pericyte-induced angiogenesis may also be a viable therapeutic target to prevent tumor growth. This has been shown with imatinib, which blocks the phosphorylation of PDGFR-β in pericytes, preventing pericyte survival and proliferation, thus reducing tumor growth (Ruan et al., 2013). Therefore, pericytes appear to be a double-edged sword in cancer and any future therapy options will require detailed understanding of their biology and function in each individual cancer environment (Meng et al., 2015).

## Current Techniques to Study Pericytes

The current focus of pericyte research is to understand their defining characteristics and roles in the healthy and diseased brain. Pericytes can be distinguished from other mural cell types by their morphology, location on the vasculature, and expression profile (Armulik et al., 2011). Recently, a two-photon imaging study of transgenic mice demonstrated the structural diversity of cortical pericytes based on morphology, vascular territory, and αSMA expression (Grant et al., 2019). Pericytes are clearly heterogeneous and the specific role of each sub-class in neurovascular function is currently unknown. New research tools such as *in vivo* two-photon microscopy and single-cell RNA sequencing as well as advancing transgenic mouse technology are being applied to answer these questions and to better understand the defining roles and characteristics of pericytes.

### Single Cell Sequencing

Given their heterogeneity in structure and function, an important future direction of pericyte research is the classification of pericyte sub-classes. Sequencing technology has advanced rapidly with researchers now examining the transcriptome, proteome, metabolome, and secretome of cells. A recent study used single-cell RNA sequencing to transcriptionally profile the principal cell types of the brain vasculature (Vanlandewijck et al., 2018). Results showed gradual arteriovenous transcriptional zonation in EC, while no such zonation or subtyping was seen within pericytes. Transmembrane transporter activity was associated with genes that were overexpressed in brain pericytes when compared with lung pericytes, providing evidence for organotypic specialization of pericytes, and suggesting that brain pericytes are directly involved in molecular transport at the BBB. Though this technique has the potential to identify pericyte subtypes, results of this particular study did not find this same zonation profile within brain pericytes. This hints that signaling from nearby cells may give rise to the different pericyte morphology that is observed along the arteriovenous axis.

### Pericyte Immunochemistry

While pericytes were initially identified based on their distinct cellular morphology, other techniques are available to identify pericytes *in vitro* and *ex vivo* using immunochemistry. Several antigen markers exist for pericytes including PDGFR-β, neural/glial antigen 2 (NG2), α-SMA, desmin, and aminopeptidase N (CD13), but all of these are also expressed by other cells, such as oligodendrocyte precursor cells and VSMC (Armulik et al., 2011). To confirm pericyte identity through their juxtaposition with capillaries, pericyte markers can be co-localized with vascular markers such as fluorescently conjugated lectins (e.g., FITC-tomato lectin) or EC markers (e.g., CD31) (Robertson et al., 2015).

### Pericytes *in vitro*

Pericytes possess the receptors and intracellular signaling machinery to respond to neurotransmitters and vasoactive mediators including noradrenaline, angiotensin-II, endothelin-1, adenosine, and EC-derived nitric oxide (Hamilton et al., 2010). *In vitro* pericyte preparations can be used to examine the molecular biology and signaling pathways controlling pericyte function. Primary pericyte cell culture using cells derived from both human and rodent tissue has shown that pericytes can contract and relax in response to vasoactive mediators (Neuhaus et al., 2017), as well as secrete cytokines relevant to neuroinflammatory responses (Rustenhoven et al., 2017). To study how pericytes interact with other neurovascular cells, specific *in vitro* models of the neurovasculature are being developed. When human brain-derived pericytes and astrocytes were cultured with human induced pluripotent stem cell-derived EC, the cultures formed vascular networks in a fibrin gel (Campisi et al., 2018). These co-cultures can be used to model the BBB and examine interactions between cells as well as transportation and diffusion dynamics of specific molecules. More recently, it was shown that brain-specific mesoderm and neural crest pericyte-like cells could be derived from human induced pluripotent stem cells (Faal et al., 2019; Stebbins et al., 2019). This introduces the possibility of culturing patient-specific pericytes, which would be a valuable tool to study how genetic disease states affect pericyte function. In combination, these techniques could enable the generation of human patient-specific isogenic 3D microfluidic systems, allowing the modeling of human neurovascular function in specific disease states to help develop therapeutics with high specificity (Stebbins et al., 2019). Another approach using *ex vivo* brain slices with the cerebrovascular architecture intact can be used to reveal molecular pathways that control pericyte functions on capillaries (Hall et al., 2014; Mishra et al., 2016).

### Pericytes *in vivo*

Transgenic mice are available with fluorescently labeled pericytes (e.g., NG2-DsRed, PDGFR-β-tdTomato), and are commonly used for imaging of pericytes *in vivo* using two-photon microscopy (Hall et al., 2014; Hartmann et al., 2015). Pericyte location on capillaries can be visualized using vascular tracers such as FITC-dextran, but a disadvantage of both NG2- and PDGFR-β reporters is that they label all mural cells which includes both pericytes and VSMC (Jung et al., 2017). Fluorescent Nissl dye Neurotrace 500/525 has been shown to preferentially bind to pericytes *in vivo* allowing the imaging of pericytes without the need for transgenic animals (Damisah et al., 2017). Mouse models of pericyte depletion have been developed (e.g., PDGFRβ^+/–^) which can lead to impaired BBB functionality (Armulik et al., 2010). In addition, taking advantage of the Cre-Lox systems, genes can specifically be knocked-in or -out within pericytes to alter their function, for example LRP-1 (Ma et al., 2018), but caution must be noted that other cell types depending on the Cre-reporter line will also be affected (e.g., VSMC and oligodendrocyte precursor cells). Optogenetic techniques, in conjunction with 2-photon imaging, have been shown to stimulate pericytes to generate a state of vessel constriction, eliciting pericyte-driven capillary diameter changes (Kisler et al., 2017a). However, this has not been consistent in all studies (Hill et al., 2015), possibly due to differences in Cre drivers used or the model and/or light source used.

## Future Directions

### Sub-Type Classification of Pericytes

In Hall et al. (2014), an important paper was published stating that pericytes can regulate CBF. Controversially, the next year a contradictory paper was published stating that VSMC, not pericytes regulated CBF (Hill et al., 2015). This disagreement appears to have arisen due to confusion about the nomenclature of pericytes (Attwell et al., 2016) and has revealed the need for a universal pericyte classification system. Several researchers have shown, using high-resolution imaging techniques, three distinct pericyte morphologies: ensheathing pericytes which cover much of the vessel surface at the arteriole-capillary junction and are thought to be contractile; mesh pericytes on the capillary and post-capillary venule which have short processes that are longitudinal and wrap around the vessel; and finally, thin-strand pericytes in the middle part of the capillary which display the traditional bump-on-a-log morphology with thin processes running along the vessel (Figure 1B; Attwell et al., 2016; Berthiaume et al., 2018). However, while single-cell sequencing could be used to show differential gene expression profiles of pericytes depending on their location along the vascular tree, a recent study showed no RNA expression changes within pericytes, and in fact pericytes had a substantially different RNA expression profile to arteriolar VSMC (Vanlandewijck et al., 2018).

### Pericyte-Specific Drug Targeting

The ability to activate or inhibit pericyte function has enormous potential to help treat the underlying cellular pathology in disease. However, specific drug delivery to pericytes is difficult considering pericytes share molecular pathways and surface receptors with other cell types. In addition, drug delivery to cerebral tissue is difficult, as the BBB allows only selective passage of cells and small molecules. While strategies to overcome this issue, such as viral vectors, exosomes, and nanoparticles, have been trialed, their use to target pericytes in the brain has been limited (Dong, 2018). One recent study demonstrated successful peptide-conjugated nanoparticle delivery of the anticancer drug docetaxel to peripheral pericytes in rats with neuroblastoma (Guan et al., 2014), while peptide-conjugated liposomal nanoparticles have also been used to effectively target pericytes and deliver the anticancer drug doxorubicin in mice with lung melanoma (Loi et al., 2010). Nanoparticle formulation with pericyte-binding peptide sequences is possible (Kang and Shin, 2016) but it has not yet been trialed in the brain. The delivery of pericyte-targeted drugs through nanoparticles could provide a novel therapeutic avenue for the treatment of many neurovascular diseases.

### Therapeutic Administration of Pericytes

Pericytes are essential to tissue repair processes; they direct angiogenesis and therefore the supply of blood to repairing tissue, as well as facilitate inflammatory responses and clear toxic substances (Ribatti et al., 2011; Jansson et al., 2014; Rustenhoven et al., 2017). It has been suggested that administration of pericytes to brain regions where injury has occurred could improve the repair process (Cheng et al., 2018). This concept has recently been examined where the implantation of pericytes into the brains of an AD mouse model improved blood flow and reduced the magnitude of Aβ pathology (Tachibana et al., 2018). The recent developments in induced human pluripotent stem cell-derived pericytes provides the possibility to create autologous pericyte cellular therapies for neurodegenerative diseases that are specific to individual patients, averting the possibility of complications such as immune rejection of cells (Faal et al., 2019; Stebbins et al., 2019). Pericytes themselves can also behave like stem cells and therefore may be reprogrammed to differentiate into other neurovascular cells such as neurons and EC (Nakagomi et al., 2015), which could alleviate neurovascular dysfunction in disease. However, caution over the administration method of pericytes, similar to stem cell therapy, must be considered when contemplating pericyte therapy for disease.

## Conclusion

While it has been nearly 150 years since the first description of pericytes, their functions in the brain, both in health and disease, are still not fully understood. Pericytes form an integral part of the NVU and act as a central mediator connecting the brain parenchyma with the vascular system, in order to meet the considerable metabolic demands of the brain. Pericytes appear to be susceptible to injury and stress and it is now recognized that pericyte degeneration can occur in multiple neurological diseases. Recent advances in technology will enable pericytologists to create a coordinated classification system for pericyte sub-types and to determine how each of these influence overall neurovascular function, in both the healthy and diseased brain. Until recently, pericytes have been largely overlooked in numerous areas of biology but their potential as either a treatment or therapeutic target to alleviate neurological disease is becoming recognized.

## Key Concepts

### Pericyte

A multi-functional vascular cell that is embedded within the capillary wall and is important in the regulation of CBF, maintenance of the BBB, vascular development and stability, and neuroinflammation. The dysfunction of pericytes in diseases such as stroke and AD has recently been highlighted.

### Neurovascular Unit (NVU)

A grouping of cells that regulate the interaction between the brain parenchyma and the blood vessels supplying it. The NVU is made up of neurons, astrocytes, microglia, pericytes, VSMC, and EC.

### Blood-Brain Barrier (BBB)

A semi-permeable membrane separating the blood from the cerebrospinal fluid, and constituting a barrier to the passage of cells, particles, and large molecules. The BBB is formed primarily by non-fenestrated endothelial cells connected by tight junctions, and is supported by pericytes and astrocyte endfeet.

### Cerebral Blood Flow (CBF)

The flow of blood throughout the brain is vital for the maintenance of brain function, as blood delivers the crucial energy substrates oxygen and glucose required for neuronal activity. CBF is tightly controlled at the molecular and cellular levels by the NVU.

### Neurological Disease

Diseases of the brain that can be caused by an acute injury, e.g., stroke, or a chronic degenerative condition, e.g., Alzheimer’s disease.

## Author Contributions

All authors listed have made a substantial, direct and intellectual contribution to the work, and approved it for publication.

## Conflict of Interest Statement

The authors declare that the research was conducted in the absence of any commercial or financial relationships that could be construed as a potential conflict of interest.

## Abbreviations

α-SMA: alpha-smooth muscle actin
Aβ: amyloid beta
AD: Alzheimer’s disease
BBB: blood-brain barrier
CBF: cerebral blood flow
EC: endothelial cells
IL-1β: interleukin-1 beta
LRP-1: low density lipoprotein receptor-related protein 1 receptor
MMP9: matrix metalloprotease 9
NG2: neural/glial antigen 2
NOTCH: neurogenic locus notch homolog protein
NVU: neurovascular unit
PDGF-BB: platelet-derived growth factor-BB
PDGFR-β: platelet-derived growth factor receptor beta
TGF-β: transforming growth factor beta
TNF-α: tumor necrosis factor alpha
VEGF: vascular endothelial growth factor
VSMC: vascular smooth muscle cell.

## References

[B1] Alarcon-Martinez L. Yilmaz-Ozcan S. Yemisci M. Schallek J. Kilic K. Can A. (2018). Capillary pericytes express alpha-smooth muscle actin, which requires prevention of filamentous-actin depolymerization for detection. *eLife* 7:e34861. 10.7554/eLife.34861 29561727 PMC5862523

[B2] Armulik A. Genové G. Betsholtz C. (2011). Pericytes: developmental, physiological and. pathological perspectives, problems, and promises. *Dev. Cell* 21 193–215. 10.1016/j.devcel.2011.07.001 21839917

[B3] Armulik A. Genové G. Mäe M. Nisancioglu M. H. Wallgard E. Niaudet C. (2010). Pericytes regulate the blood-brain barrier. *Nature* 468 557–561. 20944627 10.1038/nature09522

[B4] Attwell D. Buchan A. M. Charpak S. Lauritzen M. MacVicar B. A. Newman E. A. (2010). Glial and. neuronal control of brain blood flow. *Nature* 468 232–243. 10.1038/nature09613 21068832 PMC3206737

[B5] Attwell D. Mishra A. Hall C. N. O’Farrell F. M. Dalkara T. (2016). What is a pericyte? *J. Cereb. Blood Flow Metab.* 36 451–455. 10.1177/0271678X15610340 26661200 PMC4759679

[B6] Avolio E. Madeddu P. (2016). Discovering cardiac pericyte biology: from physiopathological mechanisms to potential therapeutic applications in ischemic heart disease. *Vascul. Pharmacol.* 86 53–63. 10.1016/j.vph.2016.05.009 27268036

[B7] Barlow K. D. Sanders A. M. Soker S. Ergun S. Metheny-Barlow L. J. (2012). Pericytes on the tumor vasculature: jekyll or hyde? *Cancer Microenviron.* 6 1–17. 10.1007/s12307-012-0102-2 22467426 PMC3601214

[B8] Bell R. D. Winkler E. A. Sagare A. P. Singh I. LaRue B. Deane R. (2010). Pericytes control key neurovascular functions and neuronal phenotype in the adult brain and during brain aging. *Neuron* 68 409–427. 10.1016/j.neuron.2010.09.043 21040844 PMC3056408

[B9] Bell R. D. Winkler E. A. Singh I. Sagare A. P. Deane R. Wu Z. (2012). Apolipoprotein E controls cerebrovascular integrity via cyclophilin A. *Nature* 485 512–516. 10.1038/nature11087 22622580 PMC4047116

[B10] Bergers G. Song S. (2005). The role of pericytes in blood-vessel formation, and. maintenance. *Neuro Oncol.* 7 452–464. 10.1215/s1152851705000232 16212810 PMC1871727

[B11] Berthiaume A.-A. Hartmann D. A. Majesky M. W. Bhat N. R. Shih A. Y. (2018). Pericyte structural remodeling in cerebrovascular health and homeostasis. *Front. Aging Neurosci.* 10:210. 10.3389/fnagi.2018.00210 30065645 PMC6057109

[B12] Cai C. Fordsmann J. C. Jensen S. H. Gesslein B. Lonstrup M. Hald B. O. (2018). Stimulation-induced increases in cerebral blood flow and local capillary vasoconstriction depend on conducted vascular responses. *Proc. Natl. Acad. Sci. U.S.A.* 115 E5796–E5804. 10.1073/pnas.1707702115 29866853 PMC6016812

[B13] Campisi M. Shin Y. Osaki T. Hajal C. Chiono V. Kamm R. D. (2018). 3D self-organized microvascular model of the human blood-brain barrier with endothelial cells, pericytes and astrocytes. *Biomaterials* 180 117–129. 10.1016/j.biomaterials.2018.07.014 30032046 PMC6201194

[B14] Cheng J. Korte N. Nortley R. Sethi H. Tang Y. Attwell D. (2018). Targeting pericytes for therapeutic approaches to neurological disorders. *Acta Neuropathol.* 136 507–523. 10.1007/s00401-018-1893-0 30097696 PMC6132947

[B15] Damisah E. C. Hill R. A. Tong L. Murray K. N. Grutzendler J. (2017). A fluoro-Nissl dye identifies pericytes as distinct vascular mural cells during in vivo brain imaging. *Nat. Rev. Neurosci.* 20 1023–1032. 10.1038/nn.4564 28504673 PMC5550770

[B16] Dong X. (2018). Current strategies for brain drug delivery. *Theranostics* 8 1481–1493. 10.7150/thno.21254 29556336 PMC5858162

[B17] Faal T. Phan D. T. T. Davtyan H. Scarfone V. M. Varady E. Blurton-Jones M. (2019). Induction of mesoderm and neural crest-derived pericytes from human pluripotent stem cells to study blood-brain barrier interactions. *Stem Cell Rep.* 12 451–460. 10.1016/j.stemcr.2019.01.005 30745035 PMC6409424

[B18] Farahani R. M. Rezaei-Lotfi S. Simonian M. Xaymardan M. Hunter N. (2019). Neural microvascular pericytes contribute to human adult neurogenesis. *J. Comp. Neurol.* 527 780–796. 10.1002/cne.24565 30471080

[B19] Grant R. I. Hartmann D. A. Underly R. G. Berthiaume A.-A. Bhat N. R. Shih A. Y. (2019). Organizational hierarchy and structural diversity of microvascular pericytes in adult mouse cortex. *J. Cereb. Blood Flow Metab.* 39 411–425. 10.1177/0271678X17732229 28933255 PMC6399730

[B20] Guan Y.-Y. Luan X. Xu J.-R. Liu Y.-R. Lu Q. Wang C. (2014). Selective eradication of tumor vascular pericytes by peptide-conjugated nanoparticles for antiangiogenic therapy of melanoma lung metastasis. *Biomaterials* 5 3060–3070. 10.1016/j.biomaterials.2013.12.027 24393268

[B21] Hall C. N. Reynell C. Gesslein B. Hamilton N. B. Mishra A. Sutherland B. A. (2014). Capillary pericytes regulate cerebral blood flow in health and disease. *Nature* 508 55–60. 10.1038/nature13165 24670647 PMC3976267

[B22] Hamilton N. Attwell D. Hall C. (2010). Pericyte-mediated regulation of capillary diameter: a component of neurovascular coupling in health and disease. *Front. Neuroenergetics* 2:5. 10.3389/fnene.2010.00005 20725515 PMC2912025

[B23] Hartmann D. A. Underly R. G. Grant R. I. Watson A. N. Lindner V. Shih A. Y. (2015). Pericyte structure and distribution in the cerebral cortex revealed by high-resolution imaging of transgenic mice. *Neurophoton* 2:041402. 10.1117/1.NPh.2.4.041402 26158016 PMC4478963

[B24] Hellstrom M. Gerhardt H. Kalen M. Li X. Eriksson U. Wolburg H. (2001). Lack of pericytes leads to endothelial hyperplasia and abnormal vascular morphogenesis. *J. Cell Biol.* 153 543–553. 11331305 10.1083/jcb.153.3.543 PMC2190573

[B25] Herland A. van der Meer A. D. FitzGerald E. A. Park T.-E. Sleeboom J. J. F. Ingber D. E. (2016). Distinct contributions of astrocytes and pericytes to neuroinflammation identified in a 3D human blood-brain barrier on a chip. *PLoS One* 11:e0150360. 10.1371/journal.pone.0150360 26930059 PMC4773137

[B26] Hill R. A. Tong L. Yuan P. Murikinati S. Gupta S. Grutzendler J. (2015). Regional blood flow in the normal and ischemic brain is controlled by arteriolar smooth muscle cell contractility and not by capillary pericytes. *Neuron* 87 95–110. 10.1016/j.neuron.2015.06.001 26119027 PMC4487786

[B27] Jansson D. Rustenhoven J. Feng S. Hurley D. Oldfield R. L. Bergin P. S. (2014). A role for human brain pericytes in neuroinflammation. *J. Neuroinflamm.* 11:104. 10.1186/1742-2094-11-104 24920309 PMC4105169

[B28] Jeong J. H. Sugii Y. Minamiyama M. Okamoto K. (2006). Measurement of RBC deformation and velocity in capillaries in vivo. *Microvasc. Res.* 71 212–217. 10.1016/j.mvr.2006.02.006 16624342

[B29] Jung B. Arnold T. D. Raschperger E. Gaengel K. Betsholtz C. (2017). Visualization of vascular mural cells in developing brain using genetically labeled transgenic reporter mice. *J. Cereb. Blood Flow Metab.* 38 456–468. 10.1177/0271678X17697720 28276839 PMC5851136

[B30] Kang E. Shin J. W. (2016). Pericyte-targeting drug delivery and tissue engineering. *Int. J. Nanomed.* 11 2397–2406. 10.2147/IJN.S105274 27313454 PMC4892861

[B31] Kim J. Basak J. M. Holtzman D. M. (2009). The role of apolipoprotein E in Alzheimer’s disease. *Neuron* 63 287–303. 10.1016/j.neuron.2009.06.026 19679070 PMC3044446

[B32] Kisler K. Nelson A. R. Montagne A. Zlokovic B. V. (2017a). Cerebral blood flow regulation and neurovascular dysfunction in Alzheimer disease. *Nat. Rev. Neurosci.* 18 419–434. 10.1038/nrn.2017.48 28515434 PMC5759779

[B33] Kisler K. Nelson A. R. Rege S. V. Ramanathan A. Wang Y. Ahuja A. (2017b). Pericyte degeneration leads to neurovascular uncoupling and limits oxygen supply to brain. *Nat. Neurosci.* 20 406–416. 10.1038/nn.4489 28135240 PMC5323291

[B34] Loi M. Marchio S. Becherini P. Di Paolo D. Soster M. Curnis F. (2010). Combined targeting of perivascular and endothelial tumor cells enhances anti-tumor efficacy of liposomal chemotherapy in neuroblastoma. *J. Control Release* 145 66–73. 10.1016/j.jconrel.2010.03.015 20346382

[B35] Ma Q. Zhao Z. Sagare A. P. Wu Y. Wang M. Owens N. C. (2018). Blood-brain barrier-associated pericytes internalize and clear aggregated amyloid-β42 by LRP1-dependent apolipoprotein E isoform-specific mechanism. *Mol. Neurodegener.* 13:57. 10.1186/s13024-018-0286-0 30340601 PMC6194676

[B36] McConnell H. L. Kersch C. N. Woltjer R. L. Neuwelt E. A. (2017). The translational significance of the neurovascular unit. *J. Biol. Chem.* 292 762–770. 10.1074/jbc.R116.760215 27920202 PMC5247651

[B37] Meng M.-B. Zaorsky N. G. Deng L. Wang H.-H. Chao J. Zhao L.-J. (2015). Pericytes: a double-edged sword in cancer therapy. *Future Oncol.* 11 169–179. 10.2217/fon.14.123 25143028

[B38] Mishra A. Reynolds J. P. Chen Y. Gourine A. V. Rusakov D. A. Attwell D. (2016). Astrocytes mediate neurovascular signaling to capillary pericytes but not to arterioles. *Nat. Rev. Neurosci.* 19 1619–1627. 10.1038/nn.4428 27775719 PMC5131849

[B39] Nakagomi T. Kubo S. Nakano-Doi A. Sakuma R. Lu S. Narita A. (2015). Brain vascular pericytes following ischemia have multipotential stem cell activity to differentiate into neural and vascular lineage cells. *Stem Cells* 33 1962–1974. 10.1002/stem.1977 25694098

[B40] Nation D. A. Sweeney M. D. Montagne A. Sagare A. P. D’Orazio L. M. Pachicano M. (2019). Blood–brain barrier breakdown is an early biomarker of human cognitive dysfunction. *Nat. Med.* 25 270–276. 10.1038/s41591-018-0297-y 30643288 PMC6367058

[B41] Neuhaus A. A. Couch Y. Sutherland B. A. Buchan A. M. (2017). Novel method to study pericyte contractility and responses to ischaemia in vitrousing electrical impedance. *J. Cereb. Blood Flow Metab.* 37 2013–2024. 10.1177/0271678X16659495 27418036 PMC5464697

[B42] Ozen I. Deierborg T. Miharada K. Padel T. Englund E. Genové G. (2014). Brain pericytes acquire a microglial phenotype after stroke. *Acta Neuropathol.* 128 381–396. 10.1007/s00401-014-1295-x 24848101 PMC4131168

[B43] Peppiatt C. M. Howarth C. Mobbs P. Attwell D. (2006). Bidirectional control of CNS capillary diameter by pericytes. *Nature* 443 700–704. 10.1038/nature05193 17036005 PMC1761848

[B44] Ribatti D. Nico B. Crivellato E. (2011). The role of pericytes in angiogenesis. *Int. J. Dev. Biol.* 55 261–268. 10.1387/ijdb.103167dr 21710434

[B45] Robertson R. T. Levine S. T. Haynes S. M. Gutierrez P. Baratta J. L. Tan Z. (2015). Use of labeled tomato lectin for imaging vasculature structures. *Histochem. Cell Biol.* 143 225–234. 10.1007/s00418-014-1301-3 25534591

[B46] Ruan J. Luo M. Wang C. Fan L. Yang S. N. Cardenas M. (2013). Imatinib disrupts lymphoma angiogenesis by targeting vascular pericytes. *Blood* 121 5192–5202. 10.1182/blood-2013-03-490763 23632889 PMC3695363

[B47] Rucker H. K. Wynder H. J. Thomas W. E. (2000). Cellular mechanisms of CNS pericytes. *Brain Res. Bull.* 51 363–369. 10.1016/s0361-9230(99)00260-9 10715555

[B48] Rustenhoven J. Jansson D. Smyth L. C. Dragunow M. (2017). Brain pericytes as mediators of neuroinflammation. *Trends Pharmacol. Sci.* 38 291–304. 10.1016/j.tips.2016.12.001 28017362

[B49] Sagare A. P. Bell R. D. Zhao Z. Ma Q. Winkler E. A. Ramanathan A. (2013). Pericyte loss influences Alzheimer-like neurodegeneration in mice. *Nat. Commun.* 4:2932. 10.1038/ncomms3932 24336108 PMC3945879

[B50] Sengillo J. D. Winkler E. A. Walker C. T. Sullivan J. S. Johnson M. Zlokovic B. V. (2013). Deficiency in mural vascular cells coincides with blood-brain barrier disruption in Alzheimer’s disease. *Brain Pathol.* 23 303–310. 10.1111/bpa.12004 23126372 PMC3628957

[B51] Shimizu F. Sano Y. Saito K. Abe M.-A. Maeda T. Haruki H. (2012). Pericyte-derived glial cell line-derived neurotrophic factor increase the expression of claudin-5 in the blood-brain barrier and the blood-nerve barrier. *Neurochem. Res.* 37 401–409. 10.1007/s11064-011-0626-8 22002662

[B52] Stebbins M. J. Gastfriend B. D. Canfield S. G. Lee M.-S. Richards D. Faubion M. G. (2019). Human pluripotent stem cell-derived brain pericyte-like cells induce blood-brain barrier properties. *Sci. Adv.* 5:eaau7375. 10.1126/sciadv.aau7375 30891496 PMC6415958

[B53] Sweeney M. D. Ayyadurai S. Zlokovic B. V. (2016). Pericytes of the neurovascular unit: key functions and signaling pathways. *Nat. Rev. Neurosci.* 19 771–783. 10.1038/nn.4288 27227366 PMC5745011

[B54] Sweeney M. D. Zhao Z. Montagne A. Nelson A. R. Zlokovic B. V. (2019). Blood-brain barrier: from physiology to disease and back. *Physiol. Rev.* 99 21–78. 10.1152/physrev.00050.2017 30280653 PMC6335099

[B55] Tachibana M. Yamazaki Y. Liu C.-C. Bu G. Kanekiyo T. (2018). Pericyte implantation in the brain enhances cerebral blood flow and reduces amyloid-beta pathology in amyloid model mice. *Exp. Neurol.* 300 13–21. 10.1016/j.expneurol.2017.10.023 29106980 PMC5745278

[B56] Underly R. G. Levy M. Hartmann D. A. Grant R. I. Watson A. N. Shih A. Y. (2017). Pericytes as inducers of rapid, matrix metalloproteinase-9-dependent capillary damage during ischemia. *J. Neurosci.* 37 129–140. 10.1523/JNEUROSCI.2891-16.2016 28053036 PMC5214626

[B57] Vanlandewijck M. He L. Mäe M. A. Andrae J. Ando K. Del Gaudio F. (2018). A molecular atlas of cell types and zonation in the brain vasculature. *Nature* 14 1–34. 10.1038/nature25739 29443965

[B58] Wang S. Voisin M.-B. Larbi K. Y. Dangerfield J. Scheiermann C. Tran M. (2006). Venular basement membranes contain specific matrix protein low expression regions that act as exit points for emigrating neutrophils. *J. Exp. Med.* 203 1519–1532. 10.1084/jem.20051210 16754715 PMC2118318

[B59] Wilhelmus M. M. M. Otte-Holler I. van Triel J. J. J. Veerhuis R. Maat-Schieman M. L. C. Bu G. (2007). Lipoprotein receptor-related protein-1 mediates amyloid-beta-mediated cell death of cerebrovascular cells. *Am. J. Pathol.* 171 1989–1999. 10.2353/ajpath.2007.070050 18055545 PMC2111121

[B60] Winkler E. A. Sagare A. P. Zlokovic B. V. (2014). The pericyte: a forgotten cell type with important implications for Alzheimer’s disease? *Brain Pathol.* 24 371–386. 10.1111/bpa.12152 24946075 PMC4423607

[B61] Xing C.-Y. Tarumi T. Liu J. Zhang Y. Turner M. Riley J. (2017). Distribution of cardiac output to the brain across the adult lifespan. *J. Cereb. Blood Flow Metab.* 37 2848–2856. 10.1177/0271678X16676826 27789785 PMC5536794

[B62] Yemisci M. Gursoy-Ozdemir Y. Vural A. Can A. Topalkara K. Dalkara T. (2009). Pericyte contraction induced by oxidative-nitrative stress impairs capillary reflow despite successful opening of an occluded cerebral artery. *Nat. Med.* 15 1031–1037. 10.1038/nm.2022 19718040

[B63] Zimmermann K. W. (1923). Der feinere bau der blutcapillaren. *Z. Anat. Entwicklungsgesch.* 68 29–109. 10.1007/bf02593544

